# Overview of clinical outcome measures used in glaucoma randomised controlled trials (RCTS)

**DOI:** 10.1186/1745-6215-14-S1-P76

**Published:** 2013-11-29

**Authors:** Rehab Ismail, Augusto Azuara-Blanco, Craig Ramsay

**Affiliations:** 1 Health Services Research unit, University of Aberdeen, Aberdeen, UK; 2 Centre for Vision and Vascular Science, Queen’s University Belfast, Belfast, UK

## Background

In clinical trials the selection of appropriate outcomes is crucial to the assessment of whether one intervention is better than another. Glaucoma is a chronic eye disease and the leading cause of irreversible blindness in the world. A variety of outcomes has been used and reported in glaucoma RCTs.

## Objectives

The purpose of this review is to identify different clinical outcome measures used in glaucoma RCTs between January 2006 and March 2012.

## Methods

A systematic review was conducted using standard methodology. We searched for RCTs in glaucoma published in English with no restrictions on the population type or size, or applied interventions. All clinical outcomes were included. Patient-reported, pharmacokinetic and economic outcomes were excluded.

## Results

The search strategy identified 4288 potentially relevant abstracts. There were 315 publications retrieved, of which 233 RCTs were included. A total of 967 clinical measures were reported. There were large variations in the definitions used to describe different outcomes and their measures. Intraocular pressure (IOP) was the most commonly reported outcome (used in 201 RCTs, 86%) with a total of 422 measures (44%). Amongst the IOP-related measures, the most commonly used was mean IOP (n=143, 15% of all measures). Safety outcomes were commonly reported, in 145 RCTs (62%) whereas visual field outcomes were utilized in 38 RCTs (16%).

## Conclusions

There is a large variability in clinical outcomes used for glaucoma RCTs and in the way each outcome is reported. This lack of standardisation may impair the ability to evaluate the evidence of glaucoma interventions.

